# Chitosan Based MicroRNA Nanocarriers

**DOI:** 10.3390/ph15091036

**Published:** 2022-08-23

**Authors:** Hussein H. Genedy, Thierry Delair, Alexandra Montembault

**Affiliations:** Université de Lyon, CNRS, Université Claude Bernard Lyon 1, INSA Lyon, Université Jean Monnet, UMR 5223, Ingénierie des Matériaux Polymères, CEDEX, 69629 Villeurbanne, France

**Keywords:** Chitosan, polysaccharide, polymer, nanoparticle, polyplex, microRNA

## Abstract

Vectorization of microRNAs has shown to be a smart approach for their potential delivery to treat many diseases (i.e., cancer, osteopathy, vascular, and infectious diseases). However, there are barriers to genetic in vivo delivery regarding stability, targeting, specificity, and internalization. Polymeric nanoparticles can be very promising candidates to overcome these challenges. One of the most suitable polymers for this purpose is chitosan. Chitosan (CS), a biodegradable biocompatible natural polysaccharide, has always been of interest for drug and gene delivery. Being cationic, chitosan can easily form particles with anionic polymers to encapsulate microRNA or even complex readily forming polyplexes. However, fine tuning of chitosan characteristics is necessary for a successful formulation. In this review, we cover all chitosan miRNA formulations investigated in the last 10 years, to the best of our knowledge, so that we can distinguish their differences in terms of materials, formulation processes, and intended applications. The factors that make some optimized systems superior to their predecessors are also discussed to reach the highest potential of chitosan microRNA nanocarriers.

## 1. Introduction

**Figure 1:**
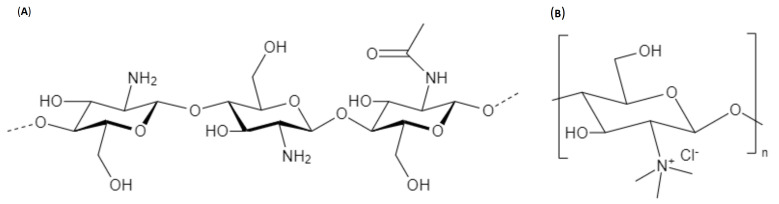
Chemical structure of (**A**) Chitosan (CS) and (**B**) Tri Methyl Chitosan (TMC).

Genetic material delivery has always been a dreadful challenge. In the last decade, non-viral vectors have shown great promise as safer alternatives to viral vectors for gene delivery. Non-viral vectors include lipidic and polymeric systems [1]. Cationic polymers were of special interest for their ability to complex genetic material readily. One of the most important cationic polymers known is chitosan since it is a natural, biocompatible, biodegradable, and generally recognized as safe (GRAS) by the FDA [2]. Chitosan is a unique polysaccharide copolymer of N-acetyl-D-glucosamine and D-glucosamine linked by a β-(1→4) glycosidic linkage (Figure 1). It is readily derived from N-deacetylation of chitin which is abundant in the exoskeletons of crustaceans (shrimps, crabs, etc.) and the endoskeletons of cephalopods (squids) and is also found in many other species such as mollusks, insects, and fungi [3]. Depending on the duration and intensity of the deacetylation process, the amount of remaining N-acetyl-D-glucosamine moieties (called the degree of acetylation, DA) varies. This parameter, along with the degree of polymerization (DP) or in other words its molar mass (Mw), should be well controlled, as many physicochemical and even biological properties are impacted by the DA and Mw of chitosan [4]. Chitosan NPs have shown potential as highly effective and safe vectors for in vivo delivery of non-coding RNAs when formulae have been optimized [5]. A chitosan based commercial gene transfection reagent (Novafect) has also been developed by Novamatrix, Norway as a non-viral carrier for gene therapy [6].

Regulatory non-coding RNAs (ncRNAs) is a term commonly used for RNA not encoding a protein yet affecting gene regulation. Classified by their average size, regulatory ncRNAs are either small non-coding RNAs (sncRNAs) typically less than 200 nt long, or lncRNAs longer than 200 nt. SncRNAs are classified into microRNA (miRNA), small interfering RNAs (siRNAs), and piwi-interacting RNAs (piRNAs) which are a class of animal specific sncRNAs that were named after PIWI proteins [7]. Both miRNAs and siRNAs have critical roles in controlling gene regulation. A lot of research has been carried out on their use for treatment of many diseases, such as cancer, osteopathy, infections, and cardiovascular diseases. Although siRNAs and miRNAs have many common features, being short double-stranded RNA molecules prompting gene silencing at the post-transcriptional level by targeting and subsequent cleavage of messenger RNA, their specific mechanisms of action and possible therapeutic applications remain different (Table 1). The major difference between them is that while siRNA is specific to just one target mRNA, miRNA has many. Hence, their therapeutic applications eventually differ [8].

**Figure 2:**
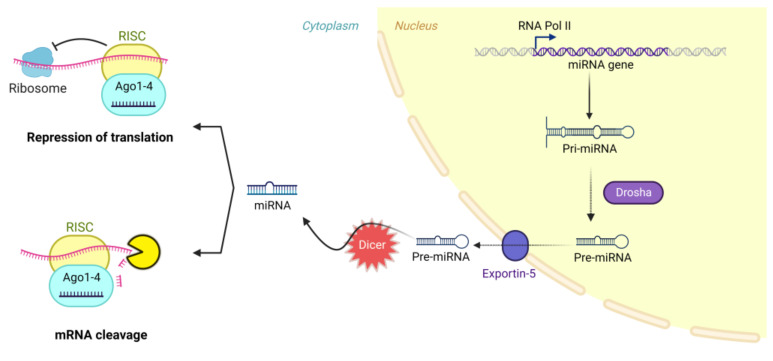
RNA interference mechanism by miRNA. Created with BioRender.com.

Both agents induce gene silencing through activation of RNA induced silencing complex (RISC). That leads to enzymatic cleavage of target mRNA in case of siRNA due to definite complementation of siRNA to its target. However, unlike siRNA, miRNA only achieves partial complementation to its target mRNA which makes it able to regulate expression of multiple targets (Figure 2) [8,9]. MiRNAs have been proven to have a role in all physiological and pathophysiological mechanisms in our bodies, which makes them groundbreaking for many therapeutic applications [10].

As a result of their gene silencing mechanisms, miRNAs hold great potential for biomedical applications, but their lack of specificity remains a challenge. Moreover, using them solely as therapeutic agents faces extracellular and intracellular barriers [11]. The extracellular barrier would be the extracellular environment containing nucleases ready to cleave any RNA therapeutic and the reticuloendothelial system RES that will mark any foreign substance detected, deeming the half-life of a therapeutic ncRNA to about 10 min.

Then, if this agent was protected either by chemical modification or vectorization, it would face intracellular barriers of cellular uptake and intracellular trafficking. As RNA interference (RNAi) agents are negatively charged, they are repelled, when administered solely, by a counter negative charge of plasma membranes compromising their uptake. In addition, their diffusion through membranes will be impossible for their high molecular weight. To top it all, from a clinical perspective, administration of miRNA as such can induce an immune response and flu like symptoms [12,13].

Ideally, successful RNAi using a delivery system requires the nanocarrier material to be biocompatible, biodegradable, nonimmunogenic, and nontoxic and needs the nanocarrier to protect ncRNA molecules from enzymatic degradation, be of an optimum size (ideally < 200 nm), surface charge, and functionalization to avoid phagocytosis and get internalized by the cells, escape lysis in the endosome, and release ncRNA in cytoplasm [14].

Many vectorization materials, including lipidic, synthetic, and natural polymeric systems, have been studied to achieve this successful delivery of ncRNA. Among them, chitosan stands out as a suitably priced available natural biocompatible biodegradable safe polysaccharide with a positive charge readily available for interaction with negatively charged ncRNA forming nanoplexes. Moreover, chitosan’s protonated amine groups enhance passage across cell membranes and endocytosis [15].

**Figure 3:**
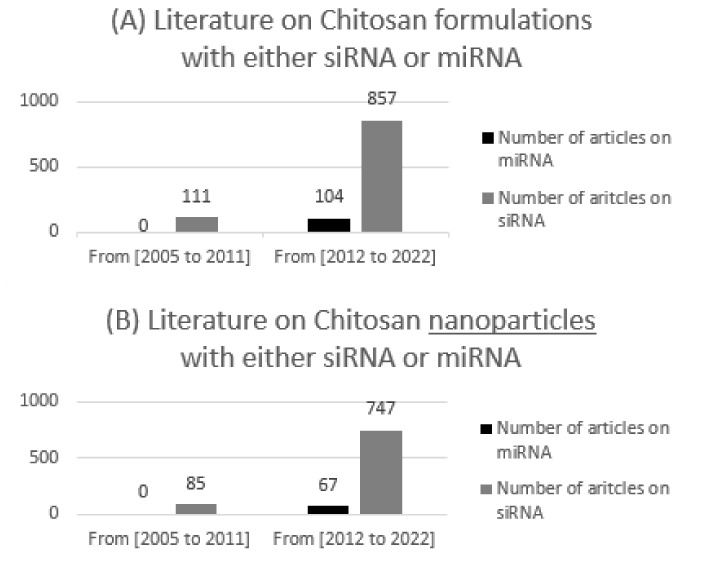
The number of publications on chitosan with either siRNA or miRNA between 2005 and 2011, and 2012–2022 using Web of Science library, with (**A**) demonstrating all formulations and (**B**) demonstrating nanoparticles only.

A lot of research has been carried out on the formulation of different forms of CS/siRNA nanoparticles and the importance of chitosan characteristics to achieving optimal (i) particle stability for ncRNA protection in biological media, (ii) colloidal stability maintaining the size of the nanoparticles avoiding aggregation, and (iii) subsequent transfection efficiency [16] but far less focus has been given to miRNA delivery using chitosan (Figure 3). To date, in 2022, only 53 research papers, all of which are covered here, tackle NPs involving both chitosan and miRNA. Indeed, both siRNA and miRNA face similar challenges which are well reviewed in other articles concerning siRNA and include poor stability in vivo, delivery challenges, and off-target effects, and similar strategies may even be investigated to optimize their delivery [14]. However, their mechanisms of actions and biomedical applications are still distinct which demands more spotlight on miRNA.

In this article, we review all chitosan-based nano-formulations made for delivery of miRNA, which include:

- Nanoparticles formulated by direct complexation between chitosan and miRNA (referred to as nano polyplexes = nanoplexes). Polyplexes are complexes made by electrostatic interaction between a cationic polymer (such as chitosan) and negatively charged genetic materials (such as miRNAs) [17].
- Nanoparticles based on the interaction between polycationic chitosan with a counter polyanion:
- Nanoparticles made by polyelectrolyte complexation of polycationic chitosan with a counter polyanion (negatively charged polymer as hyaluronic acid (HA) or dextran sulphate (DS)), adsorbing or encapsulating miRNA (referred to as CS miRNA polyelectrolyte complexes (PECs)) [18,19,20].
- Nanoparticles made by ionic gelation of polycationic chitosan with a counter polyanionic small molecule such as tripolyphosphate (TPP), adsorbing or encapsulating miRNA (referred to as CS/TPP NPs) [21].
- More complex engineered systems.

## 2. Parameters Affecting CS/MiRNA NPs

### 2.1. Chitosan Molar Mass

Chitosan molar mass is an essential parameter in the formulation of miRNA nanoparticles with optimum size and stability. CS NPs need to be stable enough to protect miRNA until it is internalized, but able to release it in cytoplasm. As studied before with CS for nucleic acid delivery, the CS chain length’s impact on NP morphological and physicochemical characteristics eventually affects their in vitro and in vivo activity [14,22].

In literature, some authors do not mention the molar mass of CS used in their studies or find it sufficient to just describe it as low or high. In this review, for uniformity, we use the same classification proposed by Ragelle et al. for CS/siRNA NPs; very high Mw would be (>300 kDa), high Mw (80-300 kDa), low Mw (10 to 80 kDa), and very low Mw CS (< 10 kDa) [14].

As concluded from work on CS siRNA NPs, CS with a very short chain length cannot condense genetic material to form intact NPs. A minimum of 15 kDa molar mass was necessary for complete binding to siRNA and therefore protection against cleavage [14]. On the other hand, a very high Mw > 300 kDa may protect the payload too much, so that it faces a challenge in gene release and subsequently less gene silencing. Only CS with molar mass 5–10 times higher than siRNA’s Mw can form compactly structured NPs of suitable size through electrostatic forces and interchain connections [23].

Most research conformed with these specifications set by experience gained with siRNA NPs. However, some exceptions can still be demonstrated. Rarely in literature has a CS of Mw more than 300 kDa been used for delivery of miRNA, however, Ning et al. reported formulation of galactosylated-CS-5-fluorouracil (GC-FU) NPs of 178.5 nm starting from 500 kDa chitosan [24]. These NPs were also loaded with liver-specific miRNA-122 to provide synergistic therapy for hepatocellular carcinoma.

#### 2.1.1. Effect of Chitosan Molar Mass on (CS/Polyanion/MiRNA Nanoparticles)

Most researchers used a Mw around 100 kDa, which demonstrated NPs of less than 200nm that were safely tolerated by cells in vitro and had a biological effect in vivo (when in vivo studies were performed). Yet, a systematic comparison of different CS molar masses has rarely been performed in the same study. Only Tekie et al. compared three different molar masses of CS (9, 18, and 45 kDa) in formulation of polyelectrolyte complexes (PECs) made with carboxymethyl dextran. PECs of 18 and 45 kDa chitosan had higher transfection efficiency, GFP expression, and reduction of cell proliferation by miR-145 when compared with PECs made of 9 kDA Mw CS [25,26].

CS/TPP NPs had a major share of interest of many scientists [9,27,28,29,30], and all of them produced intact biologically active NPs, except for McKiernan et al. as particles were not effective compared to polyethyleneimine (PEI) NPs [27], although they were well tolerated at their size of 115 nm. Deng et al., Wu et al., and Jiang et al. all added Hyaluronic acid (HA), a polyanionic biocompatible polymer, to their systems of CS-TPP NPs [28,30,31]. For Deng et al., HA addition led to a synergistic effect with DOX enhancing its antitumor effect by repressing the expression of anti-apoptosis proto-oncogene [28]. On the other hand, Wu’s NPs had a high transfection efficiency with significant enhancement of marrow mesenchymal stem cells osteogenesis [30]. Wang et al. had a similar effect of improving osteogenesis with their CS/HA system that did not include TPP at all using the same CS Mw of 100 kDa as in Wu’s work [32].

Other polyanionic polymers, like dextran and chondroitin sulfates, were also studied. Tekie et al. used a double strategy to protect their miRNA-145 by conjugating it to dextran by a disulfide bond then further electrolyte complexation with CS of 18 kDa that led to particles of 47, 132, and 272nm depending on the dextran to chitosan molar ratios used (0.2, 1 and 5 respectively) [33]. Then an aptamer was introduced as a targeting agent to increase cellular uptake. Çelik et al. introduced both HA and chondroitin, which together with CS made a triple polysaccharide system capable of protection and delivery of the miRNA to decrease mRNA levels of its target [34].

**Figure 4:**
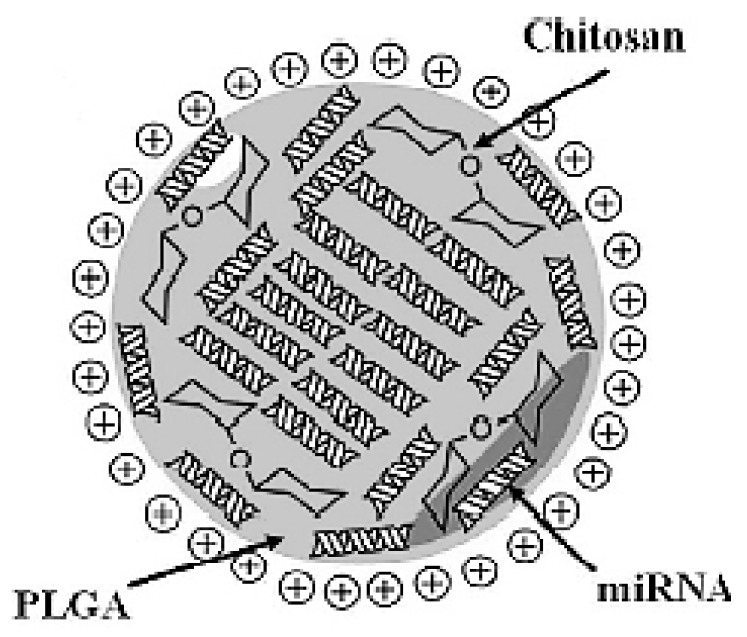
Schematic representation of miRNA-34a-loaded chitosan/PLGA nanoplexes. (Reproduced from [35]). This work is licensed under a Creative Commons Attribution 4.0 International License.

Only Cosco et al. used CS with an extremely low molar mass (<10 kDa), but they introduced the nanoprecipitation method to form PLGA/CS miRNA NPs that were intact, and of optimal size [35]. This exception may be attributed to using a totally different formulation method (nanoprecipitation) than the ionic gelation method mentioned above. PLGA was dissolved in acetone then added to acidified water containing CS, poloxamer and miR-34a all homogenized together. As PLGA has hydrophobic properties and is negatively charged, its complexation with cationic chitosan could boost the encapsulation and delivery of negatively charged hydrophilic miRNA. These CS/PLGA nanospheres, shown in Figure 4, had a mean hydrodynamic diameter of 160nm, a positive zeta potential and a high level of entrapment efficiency up to 95%. The nanospheres structure protected miRNA from degradation and allowed high transfection efficiency and a significant antitumor effect in vitro against multiple myeloma cells [35].

#### 2.1.2. Effect of Chitosan Molar Mass on Nanoplexes (Chitosan MiRNA Direct Polyplexes)

**Figure 5:**
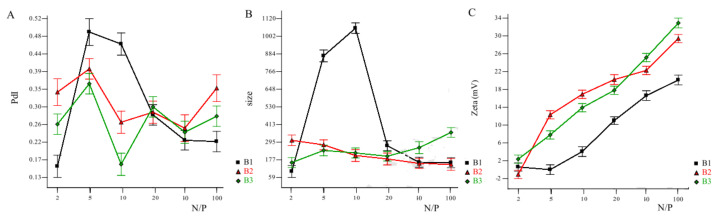
Effects of chitosan Mw and N/P ratio on (**A**) PDI, (**B**) hydrodynamic size, and (**C**) zeta potential of nanoplexes. B1 ■, B2 ▲, and B3 ♦ are chitosan Mw of 9, 18, and 45 kDa, respectively. Reprinted with permission from ref. [25]. Copyright 2015 Int. J. Biol. Macromol.

Following the same trend, all studies were conducted on molar masses of CS ranging from 20–200 kDa to form intact biologically active nanoplexes [24,25,36,37,38,39,40,41,42,43]. Exceptionally, few teams compared different molar masses in the same study, which gives more standardized results. Tekie et al. studied their nanoplexes using 9, 18, and 45 kDa CS at different N/P ratios (2, 5, 10, 20, 30, and 100) [25]. N/P (amine/phosphate) ratio is the molar ratio of polymer’s positively charged amine groups (N) to the negatively charged phosphate (P) groups of nucleic acids, and it is one of the most crucial physicochemical parameters for gene delivery, reviewed best in the work of Gary et al. and Alameh et al. [44,45]. As shown in Figure 5(B), a 9 kDa CS could not give NPs at N/P ratios of 5 and 10. Except for those two cases, all three molar masses used of CS gave nanoparticles at all used N/P ratios. However, only 9 kDa CS at N/P ratio 2 and 45 kDa CS at N/P ratio 10 gave a preferable polydispersity index PDI below 0.20. However, they indicated that their most efficient formulations were made of CS 18 and 45 kDA with N/P ratio of 40 and 100 although they showed delayed onset due to slow release [25].

**Figure 6:**
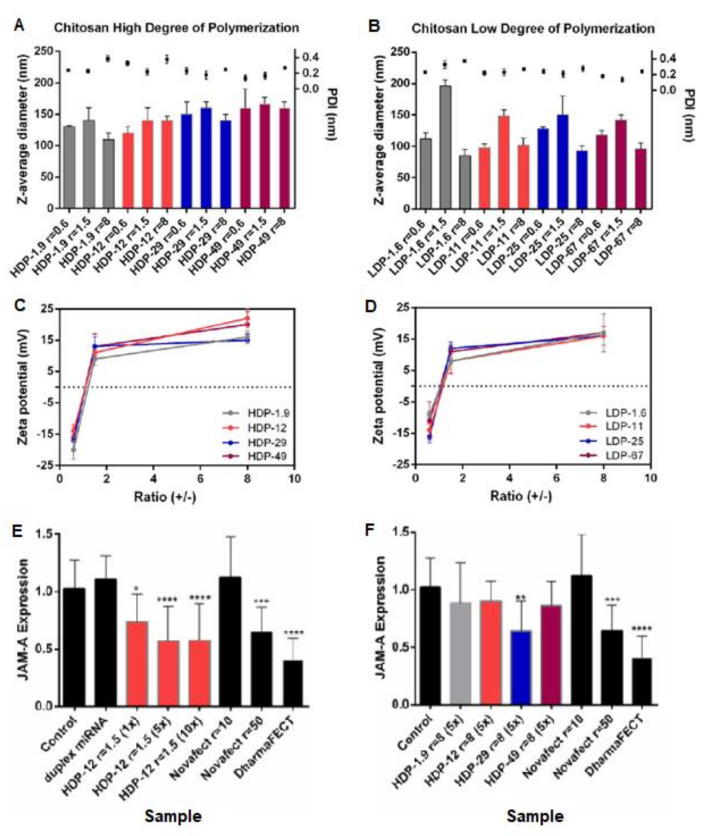
CS-miRNA polyplexes after 30 min incubation at 37o degrees. Z-average diameter in nm, polydispersity index PDI, and zeta potential ZP in samples formulated using (**A**,**C**) higher molar mass CS and (**B**,**D**) lower molar mass CS, with their respective transfection efficiency expressed as downregulation of JAM-A mRNA in MCF-7 cells: (**E**) complexes containing high molar mass CS with DA 12% (HDP-12) at (N/P) charge ratio = 1.5; and (**F**) complexes containing CS HDP-1.9, HDP-12, HDP-29, and HDP-49 at (N/P) charge ratio = 8. With HDP-DA%: Higher degree of polymerization/molar mass - degree of acetylation percent. DharmaFECT (5 µL/well) and Novafect O 25 were used as positive controls and no treatment was used as a negative control. Statistical comparisons were between each treatment and the control of untreated cells using non-parametric Kruskal–Wallis test (* *p* < 0.1; ** *p* < 0.01; *** *p* < 0.001; **** *p* < 0.0001), licensed under a Creative Commons Attribution 4.0 International License [36].

Santos-Carballal et al. studied their CS nanoplexes using two ranges of low (1–2 kDa) and relatively higher molar masses (18–26 kDa) with varying degrees of acetylation (as shown in Table 2), all produced from the same parent CS (DA = 1.5%, Mw = 543 kDa) via nitrous depolymerization and subsequent reacetylation [36]. However, a variation in molar mass can be observed. Also, an identical degree of acetylation couldn’t be achieved between comparative higher and lower molar mass couples, especially in the highest DA% couple (49% and 67%) [46,47]. These differences may have impacted the robustness of the comparison to study the effect of molar mass. All formulations led to polyplexes of average hydrodynamic diameters less than 190 nm rendering them available for cellular uptake (Figure 6). They concluded that optimal formulations were observed using CS with a molar mass of ~40 kDa, DA of 12%, and a (+/−) charge ratio of 1.5, resulting in a high transfection efficiency similar to the commercial reagents (DharmaFECT and Novafect) under a standard protocol recommended by DharmaFECT [36].

Louw et al. used three different molar masses to form CS miRNA polyplexes. A size ~ 200 nm was achieved, and particles reduced activation of microglial cells in rat model showing a very exciting potential for spinal cord injury treatment by having a transfection efficiency that is even higher than viral vectors. Yet, the definite molar mass that gave these results was never mentioned. Indeed, undisclosed details can be noticed sometimes in literature for such a crucial parameter [40].

### 2.2. Degree of Acetylation (DA) of Chitosan/Deacetylation Degree (DD)

Chitosan can be re-acetylated in solution to a required tailored DA allowing the modulation of the density of primary amines on the polymer chains [48]. Upon protonation in weakly acidic media (i.e., at a pH lower than the pKa of chitosan around ~6.4), the amine moieties provide the positive charges for complexation with the genetic material. Hence, the less DA chitosan has, the greater the density of positive charges it acquires. According to extensive research with siRNA, a high positive charge density is required to achieve strong enough electrostatic interactions with Chitosan, to obtain stable nanoparticles and to mediate endosomal escape [16]. Therefore, a DA of < 30% is required to complex siRNA and form efficient nanoplexes, have more efficient endosomal escape, and eliminate the need for a cross linker as TPP. The results of Alameh et al. in 2018 showed a significant effect of CS DA on the charge density of siRNA nanocomplexes, with optimal in vitro knock down rates using a 10 kDa CS with a 28% DA, and demonstrated a minor effect of the DP and N/P ratio on knock down efficiency [45].

Accordingly, almost all research on chitosan miRNA nanoplexes used CS with a DA ≤ 25%. However, in 2015 Santos-Carballal et al. had more efficient downregulation of the target gene using a higher DA of ~30% and proved that larger complexes were obtained with higher DAs because of extra hydrophobic chains with no positive charge to interact with miRNA [36]. Additionally, miRNA would remain protected from degradation within these chitosan polyplexes due to their enhanced conformational flexibility. Moreover, the release of the RNA, subsequent to cell uptake, was favored due to the weak interactions between the carrier and its payload, an essential factor for the effectiveness of gene therapy, which led them to investigate the binding affinity between CS and miRNA using SPR spectroscopy.

## 3. Chemical Modifications to Chitosan Reported in MiRNA Formulations

CS is readily derived by partial deacetylation of chitin which is available from many natural sources (i.e., shrimp, crab, squids, insects, and fungi) [3]. Although the source should not be a factor affecting the physicochemical properties of nanoparticles, no study has been conducted to prove so. As a matter of fact, research teams usually keep using one source they are familiar with, which is acceptable as long as a validated purification process [49,50] is implemented to make sure there are no residual impurities.

The most essential reactions needed upon handling chitosan, unless used as commercialized, are reacetylation and depolymerization. Reacetylation of the primary amine group of CS is needed to tune its charge density, solubility and other physicochemical characteristics eventually affecting NPs behavior. The reaction is usually performed in a hydroalcoholic medium ensuring homogeneous N-acetylation reaction with acetic anhydride as described by Vachoud et al. [48].

**Figure 7:**
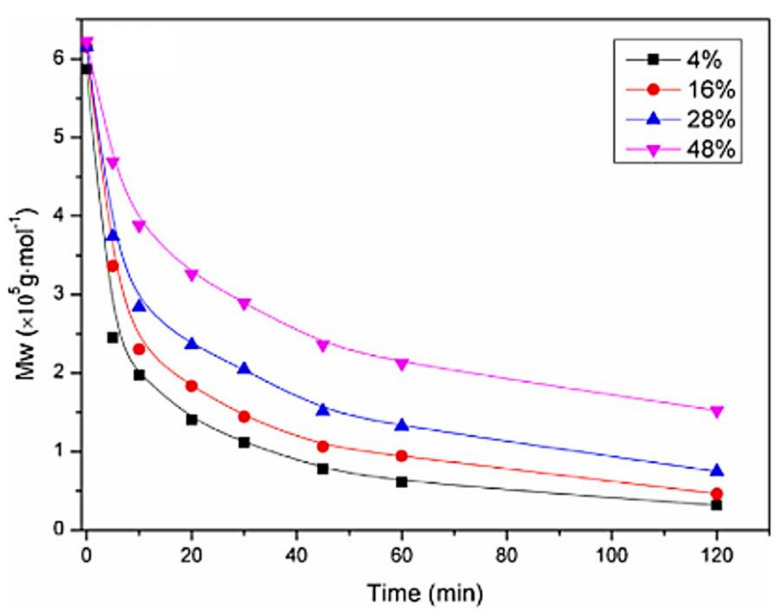
Depolymerization kinetics specifying nitrous deamination reaction duration for 4 different DA% chitosan with the same parent molar mass. Reprinted with permission from ref. [53]. Copyright 2015 Carbohydr. Polym.

Depolymerization is needed to obtain the exact lower molar mass needed to achieve desired NPs physicochemical characteristics. CS can be depolymerized chemically, enzymatically, or even by physical techniques reviewed by Younes et al. and Pandit et al. [51,52]. For the scope of our review, nitrous deamination reaction is the most well-known chemical technique to obtain CS of lower molar mass. Briefly, CS is dissolved in an acetic acid/ammonium acetate buffer of pH 4.5, then sodium nitrite is added and left stirring for a specific duration, as shown in Figure 7, needed to obtain the desired molar mass before quenching the reaction using ammonium hydroxide [36,46,47,49,53].

**Figure 8:**
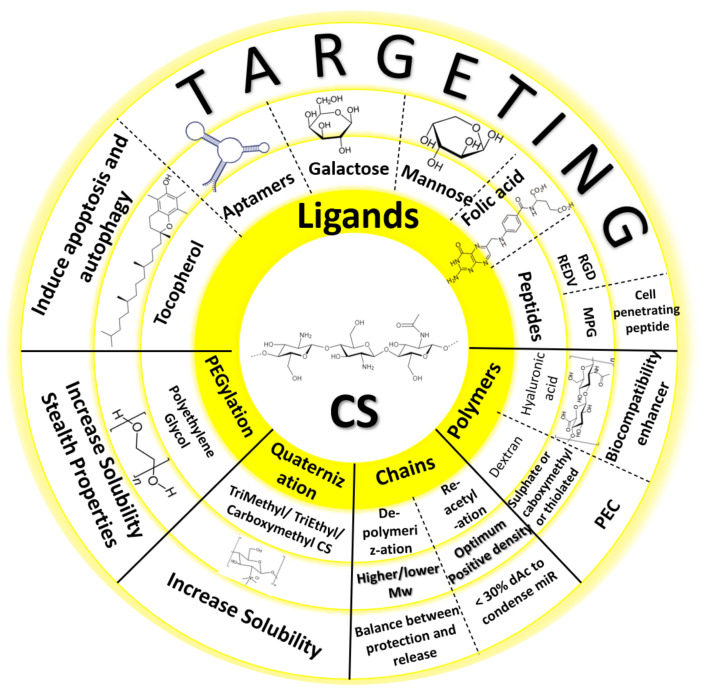
Possible chemical modifications to chitosan in miRNA formulations.

There are many possible modifications of CS to attain different characteristics (Figure 8), depending on the intended application, as it has three groups available for chemical alteration: a primary amine and two hydroxyl moieties (Figure 2). Hence, one can investigate grafting macro/small molecules to these hydroxyl groups and/or the quaternization of the primary amine [14]. Moreover, surface functionalization of chitosan NPs became a valid option to investigate active targeting ensuring better uptake and higher transfection efficiency. Ligands such as REDV peptide in the works of Zhou et al. [39,54,55], RGD peptide [56], aptamers [33,57], folic acid [58,59], galactose [24,60], mannose [61], and glutathione [50] have all been investigated within the scope of CS/miR NPs. Most studies were concerning active targeting against cancer cells, however, Pereira et al. investigated the use of lactoferrin and stearic acid to enhance the brain targeting ability of their CS nanoplexes to treat Alzheimer’s disease [62].

Many teams worked with quaternization of CS’s primary amine, which increases the solubility of CS [39,54,63,64]. They worked on Trimethyl CS (TMC) (Figure 2), except for the Safari team that worked on Triethyl CS (TEC) and N-Diethyl N-methyl (DEMC), and all teams had promising results in vitro. Zhou’s PEGylated TMC NPs enhanced endothelization in vivo when loaded in an electrospun bilayer scaffold.

**Figure 9:**
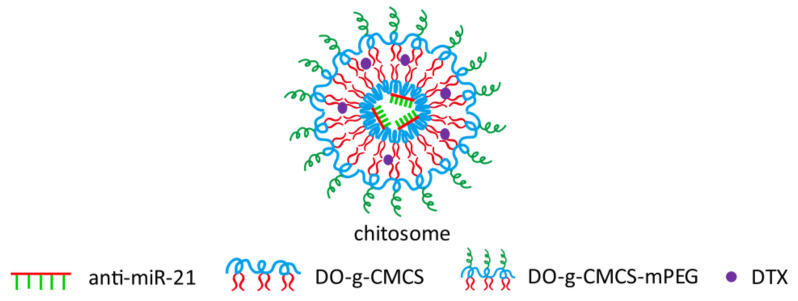
Chitosome outline containing anti-miRNA-21, dioleoyl grafted carboxymethyl CS (DO-g-CMCS), PEGylated dioleoyl grafted carboxymethyl CS (DO-g-CMCS-mPEG), docetaxel (DTX). Reprinted with permission from ref. [65]. Copyright 2020 Drug Deliv. Transl. Res.

PEGylation (i.e., attachment of polyethylene glycol) is a well-documented modification of drug nano-delivery systems. Sun et al. and Zhou et al. grafted PEG to their CS too to increase solubility and stability and avoid opsonization and subsequent rapid elimination in vivo [39,54,65,66]. PEG was usually used as a linker between CS and alkyl chains or ligands that can increase uptake of NPs by specific receptor mediated endocytosis. Sun et al. gave their CS amphiphilic properties by modifying it into PEGylated dioleoyl grafted carboxymethyl CS (DO-g-CMCS-mPEG), which was amphiphilic enough to self-assemble forming chitosomes (mimicking liposomes formed by amphiphilic lipids) (Figure 9) enabling them to co-load both hydrophobic docetaxel and hydrophilic anti-miR-21 in one NP formulation with an average particle size of 90nm [65].

## 4. Formulation Processes

The two main methods used in the literature for forming CS miRNA NPs are simple complexation and ionic gelation. Simple complexation is based on mixing miRNA with CS at definite N/P and weight ratios, both of which should be optimized with CS characteristics to form intact biologically active NPs [45]. Using this method, Santos-Carballal et al. showed that an N/P ratio of 1.5 was sufficient to achieve transfection efficiencies similar to commercial reagents (Novafect or DharmaFECT) without inducing toxicity (Figure 6) [36].

Ionic gelation on the other hand, depends on ionic interaction between the anionic charges of miRNA and the cationic charges of CS, in the presence of a crosslinking agent like tripolyphosphate TPP stabilizing and strengthening the interactions between CS and miRNA [16,67]. Katas and his team reported CS-TPP NPs to be better gene delivery vectors compared to CS/siRNA polyplexes for higher binding and loading efficiencies. CS/SiRNA interaction strength was than with simple mixing that shows siRNA and CS easily dissociating from each other. This translated into 82% gene knock down using ionic gelation compared to 51% only with simple complexation, when tested with Chinese Hamster Ovary cells (CHO K1). However, lower transfection efficiency was observed when tested with HEK 293 human kidney cells, with ionic gelation still maintaining higher transfection efficiency than simple complexation [68].

Very similarly to these methods, polyelectrolyte complexation can provide a safe and green process for formulation of miRNA NPs. A green nanomedicine process would be quick, suitably priced, and would ensure the use of only nontoxic reagents [69]. Polyelectrolyte complexes (PECs) are safe vectors spontaneously formed by mixing two water solutions of oppositely charged polyelectrolytes with no need for organic solvents, surfactants or crosslinkers [18]. It is basically the same concept as the two methods above, but it uses polyanionic polymers instead of negatively charged miRNA only in simple mixing or TPP in ionic gelation. Moreover, the formulation process is so straightforward that it only requires the addition of one polyanion solution to another polycation solution or vice versa, forming NPs instantly under low shear rate at room temperature as in “one-shot addition method” investigated by Delair et al. or dripping as formulated by Carvalho et al. [19,20]. Using this method can both increase stability of NPs and convey beneficial characteristics of the polyanion used, as for the use of HA that enhances the biocompatibility of NPs [28,32,34,70].

Three articles, by Deng et al., Wang et al. and Wu et al., have shown the use of both HA and TPP as polyanions, and both Deng and Wang et al. encapsulated their intended miRNA into CS NPs by adding it to the polyanion solution before NP formulation. Deng et al. encapsulated both doxorubicin DOX and miRNA-34a into CS NPs. These DOX-miRNA-34a co-loaded HA-CS NPs achieved 80% intracellular uptake giving a promising result toward a synergistic effect against triple negative breast cancer. On the other hand, Wang et al. added the intended miRNA to the preformulated CS/TPP/HA NPs by gentle pipetting, vortexed for a few seconds then left them for 1 h at room temperature for a complete formulation. No study was published for systemic analysis of which process is better in terms of encapsulation, release, and transfection efficiency. Hence, the manner and order of adding miRNA, either before or after NPs formulation, remains unaddressed as there are various types, molar masses, and degrees of acetylation of CS and other external factors, such as pH, ionic strength, charge ratios, and even transfection medium used, that would make a consensus on that matter almost impossible. However, Katas et al. proved that, for their system of CS glutamate-TPP, lower gene silencing was observed with systems adsorbing siRNA onto NPs (added after formulation) than systems entrapping siRNA (added to TPP solution before mixing with CS) which was attributed to more exposure to cleavage by nucleases and/or weaker binding to CS when tested with gel retardation assay [68]. Eventually, genetic material can be either (i) adsorbed or surface-associated over the NPs, (ii) complexed with the polycation forming the NP, or (iii) encapsulated inside the NP [71].

Exceptionally, Çelik et al. made quite unique triple polysaccharide nanoparticles composed of CS, HA, and Chondritin Sulfate (ChoS) with TPP again as a crosslinker. Here, miRNA was added dropwise to preformulated NPs dispersion at an N/P ratio of 30. Another exceptional formulation was achieved by Tekie et al. who made glutathione responsive CS PECs composed of chitosan complexed with a miRNA145 conjugated thiolated dextran (miR-TD) for gene delivery. A disulfide bond connected miRNA and TD, enhancing the stability and efficacy of miRNA, and allowing for glutathione responsive cleavage and release in cancer cells. Above all, a targeting aptamer was decorated onto the formed PECs that greatly improved the uptake by cancer cells [33].

No consensus can be made claiming which system is best. The three options of (i) direct CS/miRNA polyplex with simple mixing, (ii) adding TPP for the formulation with ionic gelation, or (iii) adding a polyanion (such as HA or DS) with or replacing TPP completely for totally green polyelectrolyte complexation are all available and all made sound results in their respective in vitro and in vivo tests, when performed. However, CS should be tailored differently in each method. To mix directly with miRNA, we would need lower DA (up to 30% according to Santos). Adding TPP helps when higher DA CS are used and there is not enough charge density to form intact acceptable size NPs. Exchanging TPP with HA or other polyanions adds biocompatibility characteristics to the system and removes the need for a chemical cross linker, hence the choice of the system will depend mainly on the personalized intended use of CS NPs.

## 5. Therapeutic Applications of Chitosan MiRNA Delivery Systems

There are two main therapeutic approaches dealing with miRNA therapeutics: either restoring miRNA using a miRNA mimic in diseases in which this miR is downregulated like various cancer phenotypes [72] or inhibiting the function of miRNA through miRNA antagonists (antagomirs) in diseases showing miRNA overexpression [73]. Both strategies are included in Table 3 collectively as the distinction is already clear whether miRNA was used as a therapeutic agent or a therapeutic target.

### 5.1. Cancer

**Figure 10:**
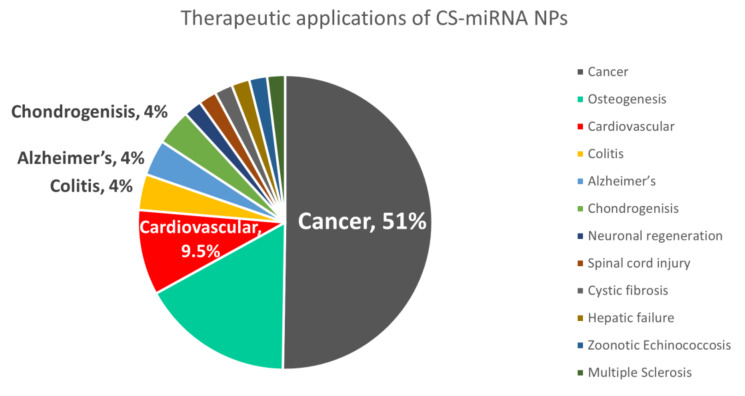
The percent of research focus on different therapeutic applications using chitosan miRNA NPs (out of 53 papers on topic from 2012 to 2022, covered in Table 3).

As demonstrated in Table 3 and Figure 10, CS miRNA NPs have been investigated mainly for cancer therapy [24,26,27,33,34,35,37,41,42,53,56,63,64,65,66,67,68]. Not only can that be attributed to the great characteristics of CS as a natural biocompatible biodegradable modifiable drug delivery system, but CS also has its own biological activities per se [4,92]. CS has been proven to possess an anticancer effect in bladder tumor cells, osteosarcoma, cervical cancer, and breast cancer cell lines [93,94,95], which may be of a synergistic anticancer effect when used in miRNA therapeutics.

As shown in the table, CS/miR nanoplexes made by simple complexation have all shown outstanding results tackling cancer with high transfection efficiencies in their respective studies. Ning and his team achieved suppressed tumor growth using their galactosylated CS with liver-specific miR 122 [24]. 

Besides nanoplexes, obtained by mere mixing of a chitosan solution and a miR solution, more sophisticated formulation processes were used, leading to chitosomes, lipopolyplexes, or nanobombs. Sun and their team achieved improved chemosensitivity of triple negative breast cancer cell line through synergy between anti-miR-21 and docetaxel in their “chitosomes” [65]. Furthermore, some research aimed at getting the best of polymer and lipids simultaneously, coming up with “lipopolyplexes” [96,97]. Recently, a near-infrared laser activated NP system, “Nano-bomb”, was developed to overcome the limitations of both materials [86]. Wang and their team developed this three-phase water-in-oil-in-water (w/o/w) structure encapsulating miR-34a indocyanine green, and ammonium bicarbonate in the inner hydrophilic phase dispersed in the polymeric hydrophobic phase of PLGA, Pluronic F127, and DPPC. The external hydrophilic phase consisted of HA and chitosan-modified-Pluronic F127. They called it a nano-bomb for the NIR laser photothermal response obtained by indocyanine green leading to the conversion of ammonium bicarbonate into gases allowing the system to escape endosome leading to efficient miR-34a delivery and gene therapy of prostate cancer stem-like cells (CSCs) [86].

### 5.2. Regenerative Medicine

Almost one third of literature on CS/miR NPs is for regenerative medicine applications. Naturally, CS has a great reputation in the regenerative medicine field as it stimulates tissue regeneration, alone or in combinations, and can convey valuable characteristics as antimicrobial and mucoadhesive properties to the systems incorporating it at definite parameters [98]. Moreover, CS has a chemical structural similarity to extracellular matrix (ECM) [99] that can be seen in the extensive research in CS/miRNA NPs for osteogenesis [30,31,32,83,84,85,87], chondrogenesis [34,89], neural tissue [76], and even blood vessels regeneration [39,54,55,64]. Jiang et al. formulated their chitosan antimir-133a/b NPs using the ionic gelation method with TPP, and hyaluronic acid then incorporated these NPs in a chitosan glycerophosphate gel making a sustained release formulation that was cytocompatible and promoted osteogenesis in vivo in a calvarial bone defect mouse model [31]. Li et al. also incorporated their CS/TPP/mir-222 NPs within silk nanofibrous scaffolds aiming at a transplantation therapy for neuronal regeneration. Their system showed good cytocompatibility and improved neural stem cells (NSCs) differentiation [76]. Zhou et al. also loaded their REDV functionalized PEGylated trimethyl chitosan miR-126 NPs into an electrospun bilayer vascular scaffold that accelerated vascular endothelial cells proliferation and could enhance reendothelialization in vivo [54].

### 5.3. Other Fields of Therapy

MiRNAs are involved in all physiological and pathophysiological mechanisms which make the opportunities for therapeutic applications endless [10]. Recently, they have received more attention for controlling inflammation [100]. Shamaeizadeh et al. investigated the use of a formulation of glutathione targeted tragacanthic acid modified CS polyplexes with miR-219a-5p for treatment of multiple sclerosis and they could prove decreased inflammation and brain cells regeneration when injecting their NPs into cuprizone MS mice model [50]. Louw et al. have also shown that their CS miR-124 polyplexes were able to reduce reactive oxygen species and TNF-α in vitro and reduce activation of microglial cells in rats in vivo aiming to treat spinal cord injury. Many more therapeutic applications are applicable depending on the miRNA chosen [40]. Cystic fibrosis [27], hepatic failure [82], and zoonotic echinococcosis [77] were also possible to investigate with CS/miR NPs

## 6. Conclusions and Prospects

Just recently in 2020, a huge surge of interest in nanomedicine has been noticed with the three phases of: waiting for a vaccine for COVID-19, the discovery of a vaccine based on nanomedicine and gene delivery, and the last phase of controversy gaining public trust with such a new system. This nanomedicine renaissance should be exploited to go further in therapeutic applications that have been overshadowed by the interest in cancer therapeutics. Fields like vaccinology, cardiovascular, hepatic, and renal diseases could use the benefits of nanomedicine. Among these nanomedicine candidates, CS NPs could provide a cheap, sustainable, stable, naturally available, and safer alternative to viral gene delivery. For it to reach the same efficiency of viral vectors, all it needs is a little push using the most recent, standardized, and consistent findings on making an efficient biologically active CS miRNA NP that is stable enough to protect miRNA from the extracellular environment, reach targeted cells, be uptaken and have weak enough binding to release miRNA in the cytoplasm for efficient gene silencing.

Achieving a systematic comparison between different factors (i.e., molar mass, degree of acetylation, N/P ratio, nanoparticle concentration, polyanions addition, and targeting moiety addition) in the same study is needed to reach a valid consensus. Recent Apliterature in the last 10 years showed more consistency and more promising results having more systemic literature-based modifications. Starting with using a molar mass of 10 kDa at least, a DA ≤ 30%, N/P ratio of 1.5–20, and PEGylation/ligand grafting when necessary is a good starting point for any future experiments. Depending on intended applications, the addition of other components to the formulations (as polyanions) can convey beneficial characteristics to the CS miRNA system. That offers a new perspective of CS and an opportunity for a theoretically old/practically new system to be exploited at its best for many therapeutic applications.

## Figures and Tables

**Table 1:** General comparison between siRNA and miRNA. [8].

	siRNA	miRNA
Composition	21–23 nucleotides duplex RNA	19–25 nucleotides duplex RNA
Complementarity to target mRNA	Complete	Partial
Target mRNA	Single	Multiple
Mechanism	Endo-nucleolytic cleavage of mRNA	- Endo-nucleolytic cleavage of mRNA - Translational repression
Clinical use	Therapeutic	- Therapeutic - Drug target - Biomarker

**Table 2:** Molar masses and DA% used in Santos-Carballal et al. studies [36].

CS sample	HDP-1.9	LDP-1.6	HDP-12	LDP-11	HDP-29	LDP-25	HDP-49	LDP-67
DA%	1.9	1.6	12	11	29	25	49	67
Mv (kDa)	26.1	1.3	25.5	1.2	20.2	1.14	18	1.95

* HDP: High degree of polymerization and LDP: Low degree of polymerization.

**Table 3:** Different CS miRNA NPs, their therapeutic applications, and in vitro and in vivo tests performed for their analysis.

Formulation	CS Characteristics	MiRNA	Application	In Vitro	In Vivo	Ref.
**(A) CS/Polyanion/MiRNA Nanoparticles**						
CS- (FD)-miRNA Fluorescein isothiocyanate Dextran	DA 3% Low M_w_ -alkyl modified CS: *N, N- Diethyl N- Methyl (DEMC) and N- Triethyl CS (TEC)	fluorescent labeled miR	Cancer	High extent transfection to human embryonic Kidney epithelial cell line (HEK 293T)		[63]
CS-TPP 115 nm	DA 10–25% CS glutamate (Protasan® UP G-113, M_w_ 160 kDa,	miR-126	Cystic fibrosis	In vitro on human cystic fibrosis bronchial epithelial cells (CFBE41o-) Safe (nontoxic to cells) but not effective		[27]
RGD labelled CS/TPP NPs	50–190 kDa Mw	miR-200	Cancer (Preventing tumor angiogenesis)	Decrease tumor cells invasion Vascular normalization Successful uptake by HeyA8 ovarian cancer cell line.		[56]
DOX-miRNA-34a co-loaded HA-CS TPP NPs	CS-HCl salt 110 kDa	miR-34a	Cancer synergistic effects	80% intracellular uptake		[28]
CS/TPP NPs	-	miR-34a	Bone-metastatic Prostate Cancer	PCa cell lines of high metastatic potential		[29]
CS/PLGA NPs 160 nm +45 mV surface charge	DA 15% low M_w_ < 10 kDa copolymer of β (1 → 4) linked 2-acetamido-2-deoxy-β -D-glucopyranose and 2-amino- 2-deoxy-β -D-glucopyranose.	miR-34a	Multiple myeloma	High transfection efficiency → By FACS analysis of SKMM1 (human multiple myleoma cell line) after 6 h treatment with the NPs	Decreased tumor size	[35]
CS/TPP NPs	CS HCl (Cl113) Mw 110 kDa	miR-199a-5p agomir	Bone regeneration	Successful transfection of cultured hMSCs	Improved bone regeneration of in rat tibia defect model.	[74]
microRNA-21-loaded CS/HA NPs	DA 10% M_w_ 100 kDa	miR-21	Enhancing osteogenesis of (hBMMSC) human bone marrow mesenchymal stem cell sheets	Higher than 90% transfection efficacy after 24 h of treatment and more than 75% on the 14th day. Significant improvement in the in vitro osteogenic differentiation (through increased calcification-related gene expression, improved production of alkaline phosphatase, more secretion of collagen, and mineralized nodule formation.		[32]
CS/TPP/HA NPs	DA 10% M_w_ 100 kDa	AntimiR-138	Improving osteogenesis	Rat bone marrow mesenchymal stem cells (MSCs) Cytocompatibility with MSCs. Around 70% transfection efficiency significantly improving MSCs osteogenesis		[30]
CS-TPP NPs	low Mw Cs	miR-34a	Cancer	Prostate tumor PC3MM2 cells		[9]
CS-carboxymethyl dextran PECs	CS 9, 18, and 45 kDa obtained by NaNO_2_ depolymerization of 400 kDa Mw CS	miR-145	Cancer	Aim to optimize PECs dissociation rate and stability Distinct proliferation reduction of MCF-7(human breast cancer cell line)		[26]
Glutathione responsive CS-thiolated dextran conjugated miRNA-145 NPs targeted with AS1411 aptamer (40–270 nm)	DA 11% 18 kDa	miR-145	Cancer	Breast cancer MCF7 cell line A significant increase of miRNA-145 in the NP -treated cells A prominent decrease of MUC1 protein after treatment A decrease in relative cell viability to 80%		[33]
CS-TPP NPs 150−200 nm	DA 15-25% M_w_ (50–190 kDa)	miR-33	Prevention and treatment of cardiovascular diseases (CVD) by delivering NPs to macrophages where they can function to regulate ABCA1 expression and cholesterol efflux, - target atherosclerotic lesions.	Transfer exogenous miRNA-33 to naive macrophages and reduce the expression of ABCA1, a potent miRNA-33 target gene, both in vitro and in vivo	Mice treated with miRNA33-chNPs showed decreased reverse cholesterol transport (RCT) to the plasma, liver, and feces. - Improvement of cholesterol efflux into the RCT pathway and ABCA1 expression when treated with CS NPs loaded with efflux-promoting miR.	[67]
Triple polysaccharide (chondroitin sulfate/hyaluronic acid/CS) nanoparticles	DA 20% 150 kDa	miR-149-5p	Chondrogenesis of human mesenchymal stem cells (osteoarthritis)	60.97% gene loading efficiency 82.98% increase in miRNA level compared to control group, inhibiting the target gene by 61.57% No toxicity to human mesenchymal stem cells Increased miRNA levels in cells Target gene inhibition Induce chondrogenic markers synthesis and responsible genes upregulation.		[34]
CS/TPP/HA NPs (100–300 nm) in CS/glycerophosphate (GP) gel	-	antimiR-133a/b	Bone regeneration	No cytotoxicity to murine bone marrow stromal cells (BMSCs) Enhanced osteogenic differentiation.	Promote osteogenesis in vivo in mouse calvarial bone defect model.	[31]
CS/TPP NPs	Medium Mw (non-specified)	miR-155-5p & AntagomiR-324-5p	Ovarian Cancer	In vitro in cell line SKOV3 Targets’ (HIF1α & GLI)1 expression was downregulated after treatment		[75]
CS/TPP NPs (350 nm) incorporated with silk fibroin (SF) nanofibrous scaffolds	CS M_w_ of 300 kDa DA 15%	miR-222	Neural stem cells (NSCs) transplantation therapy for neural tissue regeneration	Good cytocompatibility Improve NSCs neuronal differentiation.		[76]
CS/TPP NPs 150 nm	CS (Macklin)	E. multilocularis miR-4989	Treatment of Alveolar echinococcosis “Zoonotic disease”	Low cytotoxicity Strong miRNA protection No significant effects on cell proliferation nor apoptosis.	Remarkable liver tropism. UBE2N expression significantly decreased in the liver on both mRNA and protein levels.	[77]
CS/TPP NPs camouflaged with macrophage-derived exosomes [MEXO] 143.2 ± 14 nm /−10.3 ± 1.6 mV	CS HCl	miR-144/451a	Oral squamous cell carcinoma (OSCC)	MiR protection from degradation. Cytotoxic to OSCC (in UM-SCC083A and UPCI-SCC029B cell lines). Inhibition of the expression of calcium-binding protein 39 (CAB39) and migration inhibitory factor (MIF).		[78]
Folic acid targeted CS/TPP NPs ~100nm +7.3 ± 2 mV	50–190 kDa	miR-126	Lung Cancer	Significantly cytotoxic to A549 (folic acid receptor-positive lung cancer cell line) Cytocompatible with MRC5 (Normal human diploid cell line)		[58]
CS/TPP NPs 112.2 ± 2.27 nm 33.9 ± 0.9mV	50–190 kDa DA% 15–25%	miR-219	Glioblastoma	Reduced survival of human GBM cell line (U87MG) No cytotoxic effect on fibroblasts		[79]
**(B) CS-miRNA nano polyplexes (Nanoplexes)**						
CS nanoplexes: preparation via coacervation method	DA 15% Medium M_w_ CS 9, 18, and 45 kDa	miR-145	Cancer	Expression in MCF-7 breast cancer cells		[25]
CS–has-miRNA-145 nanoplexes	DA = 1.5%, M_w_ = 543 kDa modified into: [2–26 kDa] [2–67 % DA%]	miR-145	Cancer	MCF-7 breast cancer cell line. Nontoxic Determined transfection efficiency by successful downregulation of the expression of target (JAM-A) mRNA in MCF-7 cells.		[36]
Galactosylated low Mw CS (G-LMWC) nano complexes	low Mw (non-specified)	miR-16 precursors	Colitis Crohn’s diseases	MiR-16 specific upregulation of in colonic macrophages of colitic mice which significantly reduces TNF-α and IL-12p40 expression, stopping mucosal inflammation and leading to relief of colitis symptoms.		[60]
CS/pre-miRNA-29b nanoplexes~130 nm	M_w_ = 50–190 kDa	Recombinant pre-miRNA-29b	Alzheimer’s disease (AD)	Efficient delivery of the pre-miR to N2a695 (mouse neuroblasts cell line)78% decrease of hBACE1 protein expression44% decrease of Aβ42 levels		[37]
CS/miRNA-200c nanoplexes (294 nm)	DA 15–25% 75 kDa	miR-200c	Breast cancer	Reduced angiogenesis, invasion, and metastasis. Increase in apoptosis by 3.1, 1.3, and 3-fold in the MCF-7, MDA-MB-231, MDA-MB-435 breast cancer cell lines.		[38]
CS/miRNA-141 CS/miRNA-200c nanoplexes [294–380 nm]		miR-200c- and miRNA-141-	Breast cancer (Dose study)	100% transfection efficiency in the MCF-7, MDA-MB-231, and MDA-MB-435 breast cancer cell lines.		[80]
REDV peptide modified PEG trimethyl CS TMC-g-PEG-REDV/miRNA nanoplexes	DA 5% M_w_ 50 kDa modified into TMC	miR-126	Vascular endothelial cells for cardiovascular and cancer therapeutics	VECs and VSMCs (primary culture from Sprague-Dawley rat aorta, passage 3–5) MiRNA expression efficiency up to 3.4-fold compared to control group. Cytocompatibility High transfection efficiency, increase in endothelial cellular uptake, and improved VEC proliferation.		[39]
CS nanoplexes 222.0 nm	DA 5% M_w_ 30 kDa, 150 kDa and 250 kDa	miR-124	Spinal cord injury	Decreased microglial cells activation No significant decrease in viability - (Ex vivo in Neonatal rat microglia) Decrease of TNF-α and reactive oxygen species (ROS) Decreased MHC-II expression.	Decreased microglial cells activation in rat models (adult female Sprague-Dawley rats). Significant decrease in ED-1 positive macrophages in spinal cord injury	[40]
miRNA-218-loaded carboxymethyl CS-Tocopherol NPs ~110 nm	DA 10% O-carboxymethyl CS (OCMC) tocopherol polymer conjugate	miR-218	GIT stromal tumor	Human GIST cell lines (GIST882) Inhibited proliferation and exhibited superior apoptosis. Inhibited cell invasion Promoted GIST cancer cells apoptosis		[41]
GO-CS-MPG-miRNA33a/miRNA199a nanoplexesGO: Graphene oxide MPG: Cell penetrating peptide	-	miR33a/miR199a	Melanoma	Human melanoma A375 and L929 cells. Well compatible with L929 cells with low cytotoxicity at 80 mg/mL. Transfection: A375 pinocytosis of FITC-GO-CS-MPG microspheres particles could suppress melanoma A375 cells growth.	Subcutaneous tumor implantation in nude mice Significant suppression of the tumor volume	[43]
CS/miRNA-141 nanoplexes 296 nm	75 kDa DA 15–25%	miR-141	Breast cancer	MDA-MB-231 and MDA-MB-435 cells Relative miRNA expression increased by 7.5-fold compared to control group in MDA-MB-435 cells. Decrease in metastasis, VEGF and invasion. Increased apoptosis levels by 1.5- and 2.4-fold.		[42]
galactosylated-CS-5-fluorouracil (GC-FU) NPs 178.5 nm	DA 4% M_w_ 500 kDa	Liver-Specific miR-122	Synergistic Therapy for Hepatocellular Carcinoma	~95% transfection efficiency Enhanced blood and salt stability, Marked induction of HepG2 cells apoptosis, cell cycle arrest. Decreased HCC cells proliferation, migration, and invasion Downregulation of Bcl-2 and ADAM17 expression in HepG2 cells	By subcutaneous xenografts in the armpit of BALB/c nude mice. Suppressed tumor growth. High biocompatibility. No weight loss nor alteration of liver, heart, and kidney functions.	[24]
CS/miR-34a nanoplex ~135 nm +34 mV	Mw 50 kDa; 15–25% DA	miR-34a	Breast Cancer	Increased relative expression level of the miR in MDA-MB-231 cell line. Nontoxic to HUVECs normal cells. Inhibited growth, migration, and invasion of MDA-MB-231.		[81]
mannose-modified trimethyl CS NPs [MTC-miR146b] NPs 213.6 nm +28.3 mV	Trimethyl CS TMC quaternization degree =50% Mw 100 kDa	miR146b	Ulcerative Colitis	Strongly inhibited M1 macrophage activation leading to suppression of pro-inflammatory cytokines induction. Overexpression in bone marrow-derived macrophages [BMDMs]. Improvement of colon epithelial cells proliferation.	After mucosal damage, NPs restored body weight and function of mucosal barrier. Protected miR-146b-deficient mice from dextran sodium sulphate [DSS] injury and consequent cancer	[61]
Lactoferrin-Stearic Acid-Modified-CS Polyplexes CS-SA-Lf/pre-miR-29b 325.60 ± 30.99 nm +33.50 ± 2.31 mV	190–310 kDa; 15–25% DA	Pre-miR-29b	Alzheimer’s disease (AD) treatment	Non-toxic. Enhanced brain-targeting ability Suppression of BACE1 mRNA involved in AD. NPs with two ligands had higher internalization than those with one or no ligands, in neuronal cells. Efficient crossing of BBB		[62]
Glutathione (Glu) targeted tragacanthic acid (TA)- CS Polyplexes	12 kDa CS (Obtained by NaNO_2_ depolymerization from 50 kDa)	miRNA-219a-5P	Multiple sclerosis (MS) Brain delivery	In vivo injected into the cuprizone model of MS mice Decreased inflammation and increased brain cell regeneration Overexpression of miR-219, upregulation of crystallin alpha B, and downregulation of apolipoprotein E.		[50]
**(C) Scaffolds and other systems**						
nanostructured amphipathic carboxymethyl–hexanoyl CS (CHC)		miR-122 liver specific	Stem cell therapy for hepatic failure	High transfection efficiency. Facilitated differentiation of human dental. pulp-derived iPSCs (DP-iPSCs) into iPSC-Heps (miRNA122-iPSC-Heps) with functional mature hepatocytes. Hepatoprotection in fulminant hepatic failure experimental model.		[82]
Scaffolds containing CS/carboxymethyl cellulose/mesoporous wollastonite	Low Mw CS	_	Bone tissue engineering	Human osteoblastic cells (MG-63) Significant enhancement in protein adsorption and biomineralization properties. Cytocompatibility with human osteoblastic cells. Osteogenic potential proved by calcium deposition and expression of an osteoblast specific miR.		[83]
Microarc-oxidized titanium surfaces decorated with miR-21-loaded CS/HA NPs 160 ± 10 nm, positive ZP	DA 10% M_w_ 100 kDa	miR-21	(Dental) promote the osteogenic differentiation of human bone marrow mesenchymal stem cells	Human bone marrow mesenchymal stem cells (hBMMSCs). Transfection efficiency of more than 90%. Nontoxic		[84]
TMC-g-PEG-REDV/miRNA loaded in an electrospun bilayer vascular scaffold	DA 5% M_w_ ∼50 kDa then modified into TMC	miR-126	Vascular endothelial cells for blood vessel regeneration	Significant down regulation of SPRED-1 gene expression in VECs on the third day of treatment. The membranes release miR accelerating VEC proliferation within 9 days.	Replacement of rabbit carotid artery for 8 weeks suggested that the PELCL-TPRm scaffold loaded with miRNA could enhance endothelialization in vivo	[54]
In situ injectable miR-activated matrix miR-loaded-NPs entrapped into an O-carboxymethyl CS (CMCS) network	Carboxymethylation was 80% (4 kDa)	miR-21	Bone regeneration/repair.	Human umbilical cord mesenchymal stem cells (hUMSCs). High uptake efficiency (61.6%). Significantly promoted osteogenic differentiation of (hUMSCs) as evidenced by upregulation of osteogenic markers (alkaline phosphatase ALP and runt-related transcription factor (RUNX-2).	60 male Sprague-Dawley (SD) rats Promoted bone formation (osteogenesis) significantly (2.4-fold) compared to controls	[85]
A NIR laser activated Lipopolyplex “Nano-bomb” developed as w/o/w system with miR-34a in the inner hydrophilic phase dispersed in the hydrophobic phase of PLGA, Pluronic F127 & DPPC in an external hydrophilic phase of HA and CS-modified-Pluronic F127.	-	miR-34a	Prostate Cancer	In 2D cultured PC-3 cancer cells and 3D CSCs-enriched prostaspheres. Minimal cytotoxicity	Excellent anticancer safety and efficacy in mice Ability of targeting Significant reduction of tumor volume	[86]
CS/nano-hydroxyapatite/nano-zirconium-dioxide scaffolds	-	miR-590-5p	Bone regeneration	Mouse mesenchymal stem cells (C3H10T1/2) Stimulated mMSCs differentiation towards osteoblasts - Biocompatible nature		[87]
miR-126 encapsulated by REDV peptide-modified TMC-g-PEG loading PELCL/PCL-REDV electrospun membranes	TMC-g-PEG-REDV synthesized by a bifunctional PEG linking TMC with a short peptide REDV	miR-126	Cardiovascular preventing thrombosis	Enhanced VEC adhesion and proliferation Downregulation of SPRED-1 gene expression.		[55]
TMC-g-PEG-VAPG/miRNA-145 NPs in electrospun membranes	DA 10% M_w_ 50 kDa TMC	miR-145	target-regulating vascular SMCs - small-diameter blood vessel regeneration.	Low cytotoxicity Enhance cellular uptake in SMCs Inhibit the excessive proliferation and intimal hyperplasia Maintain SMCs in controlled proliferation		[64]
supramolecular self-assembled “chitosome” with anti-miRNA & docetaxel 90 nm	Carboxymethyl CS (CMCS) M_w_ 10,000 kDa further modified into DO-g-CMCS-mPEG2000	hydrophilic anti-miR-21	triple negative breast cancer (TNBC)	Significant improvement in transfection efficiency and stability against enzymatic cleavage. Improved chemosensitivity of TNBC cells (cell line of MDA-MB-231) through synergistic mechanisms.		[65]
polyelectrolyte multilayers (PEMs) of (CS-miRNA) complex & Na-hyaluronate (HA) on microarc-oxidized (MAO) microporous TiO2 surfaces.	Mw 100 kDa 5% DA	antimiR-138	Bone regeneration “Advanced implants to promote osseointegration”	Significant knockdown of miR-138 Nontoxic Increased osteogenic differentiation of MSCs (increased alkaline phosphatase, collagen production, and ECM mineralization)	Improved osseointegration in rat femur model	[88]
A phosphorylatable nuclear localization signal-linked nucleic kinase substrate short peptide (pNNS) conjugated to CS (pNNS-CS)	_	miRNA-140	Treating cartilage defects	Transfected into chondrocytes. High miRNA-140 expression levels. Improved proliferation of chondrocytes. Improved production of glycosaminoglycan GAG - Higher levels of TIMP-1, aggrecan and collagen type II alpha 1 chain. Suppression of NO, disintegrin and (MMP)-13 levels.	Target: improve repair of full-thickness cartilage defects in a rabbit model - Injected into the knee joint cavity - High miRNA-140 expression levels - Reduced synovial fluid GAG and NO levels. - Reduced cartilage ADAMTS-5 and MMP-13 levels. - Increased COL2A1, ACAN, and TIMP-1 levels - Decreased cartilage Mankin score in transgenic group.	[89]
Au-IONPs coated with β-cyclodextrin-CS (CD-CS) hybrid polymer to co-load both miR-100 and antimiR-21. With PEG-T7 peptide as a surface functionalization.	β-cyclodextrin-CS (CD-CS) hybrid polymer	miR-100 and AntimiR-21	Cancer (Glioblastoma) Theranostics	Enhanced cellular uptake of miR-100 and AntimiR-21 in GBM. Pre-sensitized GBM Cells to (TMZ) chemotherapy. Synergistic effect activating apoptotic signaling pathway in GBM cells leading to improvement of TMZ therapy.	- Intranasal delivery to U87-MG GBM cell-derived orthotopic xenograft mice models. - Cy5-miRNAs accumulated efficiently in mice - Significant increase in survival of mice co-treated with NPs and systemic TMZ.	[90]
polyglutamate PGA grafted CS core, dextran sulfate as a complexing agent, poly-ethylene-imine shell decorated with folic acid. 123 ± 5 nm-36 ± 1 mV ZP	-	miR-34a in the shell and cytotoxic peptides by the core.	Cancer	No cytotoxicity A synergistic effect leading to multiple cell death in chemoresistance human breast adenocarcinoma cell line, MDA-MB-231. - enhanced smart death induction by 54%.		[91]
DOX/Mesoporous Silica NPs coated with CS to electrostatically attach antimir-21 and Aptamer AS1411	50 kDa –190 kDa 15-25% DA	antimiR-21	Cancer	Cytotoxic to nucleolin positive (C26, MCF-7, and 4T1).	Anticancer activity in C26 colorectal cancer bearing mice	[57]
FA functionalized CS-coated Zn-MOF nanocomposites ~ 200nm	50–190 kDa Mw	LNA-antisense miR-224	Colon Cancer	In vitro on HCT116 (FA receptor-positive colon cancer cell line) and CRL1831 (normal colon cell line) Decreased cell viability Upregulated apoptotic genes expression Upregulated autophagy-related genes expression		[59]

## Data Availability

Data sharing not applicable.
